# Diagonal Earlobe Crease is a Visible Sign for Cerebral Small Vessel Disease and Amyloid-β

**DOI:** 10.1038/s41598-017-13370-8

**Published:** 2017-10-17

**Authors:** Jin San Lee, Seongbeom Park, Hee Jin Kim, Yeshin Kim, Hyemin Jang, Ko Woon Kim, Hak Young Rhee, Sung Sang Yoon, Kyoung Jin Hwang, Key-Chung Park, Seung Hwan Moon, Sung Tae Kim, Samuel N. Lockhart, Duk L. Na, Sang Won Seo

**Affiliations:** 1 Department of Neurology, Samsung Medical Center, Sungkyunkwan University School of Medicine, Seoul, 06351 Korea; 2 Neuroscience Center, Samsung Medical Center, 06351 Seoul, Korea; 3 Department of Neurology, Kyung Hee University Hospital, Seoul, Korea; 4 Department of Neurology, Chonbuk National University Hospital, Jeonju, Korea; 5 Department of Neurology, Kyung Hee University Hospital at Gangdong, Kyung Hee University School of Medicine, Seoul, Korea; 6 Departments of Nuclear Medicine, Samsung Medical Center, Sungkyunkwan University School of Medicine, Seoul, 06351 Korea; 7 Department of Radiology, Samsung Medical Center, Sungkyunkwan University School of Medicine, Seoul, 06351 Korea; 8 Helen Wills Neuroscience Institute, University of California Berkeley, Berkeley, CA 94720 USA; 9 Department of Internal Medicine, Division of Gerontology and Geriatric Medicine, Wake Forest School of Medicine, Winston-Salem, NC 27157 USA; 10 Department of Health Sciences and Technology, SAIHST, Sungkyunkwan University, Seoul, 06351 Korea; 11 Clinical Research Design and Evaluation, SAIHST, Sungkyunkwan University, Seoul, 06351 Korea

## Abstract

We investigated the frequency and clinical significance of diagonal earlobe crease (DELC) in cognitively impaired patients using imaging biomarkers, such as white matter hyperintensities (WMH) on MRI and amyloid-β (Aβ) PET. A total of 471 cognitively impaired patients and 243 cognitively normal (CN) individuals were included in this study. Compared with CN individuals, cognitively impaired patients had a greater frequency of DELC (OR 1.6, 95% CI 1.1–2.2, *P* = 0.007). This relationship was more prominent in patients with dementia (OR 1.8, 95% CI 1.2–2.7, *P* = 0.002) and subcortical vascular cognitive impairment (OR 2.4, 95% CI 1.6–3.6, *P* < 0.001). Compared with Aβ-negative cognitively impaired patients with minimal WMH, Aβ-positive patients with moderate to severe WMH were significantly more likely to exhibit DELC (OR 7.3, 95% CI 3.4–16.0, *P* < 0.001). We suggest that DELC can serve as a useful supportive sign, not only for the presence of cognitive impairment, but also for cerebral small vessel disease (CSVD) and Aβ-positivity. The relationship between DELC and Aβ-positivity might be explained by the causative role of CSVD in Aβ accumulation.

## Introduction

Diagonal ear lobe crease (DELC) is a wrinkle-like line extending diagonally from the tragus and across the lobule to the rear edge of the auricle of the ear^1,2^. Previous studies have shown that the presence of DELC predicts coronary artery disease (CAD)^1^. Although some studies argue that DELC is a phenomenon of skin aging^3–5^, recent studies have shown that DELC is associated with the development of cardiometabolic syndrome^6–9^, independent of age, sex, or race^10^.

Alzheimer’s disease (AD) and cerebral small vessel disease (CSVD) are the two most prevalent causes of cognitive impairment in the elderly^11^. Recent advances in neuroimaging techniques have made it possible to diagnose AD and CSVD more accurately during a patient’s lifetime. However, as these imaging assessments are expensive and time-intensive, it is important to first screen for people likely to have these diseases. In particular, it would be helpful if visible signs of underlying disease processes could be observed on the surface of the body. Further, AD and CSVD share common risk factors such as age and cardiometabolic syndrome. We therefore hypothesized that DELC would be a visible sign for AD and CSVD.

In this study, we evaluated the frequency of DELC in 471 cognitively impaired patients and 243 cognitively normal (CN) individuals. We further investigated the associations between DELC and imaging biomarkers, such as white matter hyperintensities (WMH) on brain magnetic resonance imaging (MRI) and amyloid-β (Aβ) on positron emission tomography (PET).

## Results

### Demographic and clinical characteristics

Demographic and clinical characteristics of the study participants according to the presence of DELC are presented in Table 1. The frequency of DELC in cognitively impaired patients was 59.2%, while it was 44.0% in CN individuals. The DELC group was older than the No DELC group for both CN individuals and cognitively impaired patients. In CN individuals, the DELC group had more frequent diabetes mellitus than the No DELC group (*P* = 0.017). In cognitively impaired patients, the DELC group had more frequent hypertension (*P* = 0.013) and history of stroke (*P* = 0.014) than the No DELC group.

**Table 1:** Demographic and clinical characteristics of the study participants according to the presence of DELC.

	CN (N = 243)			Cognitively impaired (N = 471)		
	No DELC	DELC	*P*-value	No DELC	DELC	*P*-value
Total, N	136 (56.0%)	107 (44.0%)		192 (40.8%)	279 (59.2%)	
Age, years	69.4 (6.8)	72.6 (6.9)	<0.001	71.4 (9.2)	74.0 (7.6)	0.001
Male	32 (23.5%)	36 (33.6%)	0.081	72 (37.5%)	120 (43.0%)	0.232
Education, years	10.7 (4.7)	9.9 (5.2)	0.198	11.2 (5.1)	10.4 (5.6)	0.155
*APOE* genotype^†^						
*APOE* ε2 present	18 (14.6%)	14 (14.9%)	0.957	14 (8.1%)	27 (9.9%)	0.530
*APOE* ε4 present	20 (16.3%)	18 (19.1%)	0.579	72 (41.6%)	101 (36.9%)	0.315
MMSE	28.3 (2.1)	28.2 (1.8)	0.810	23.2 (5.4)	22.4 (5.4)	0.129
CVD risk factors						
Hypertension	59 (43.4%)	54 (50.5%)	0.272	99 (51.6%)	176 (63.1%)	0.013
DM	18 (13.2%)	27 (25.2%)	0.017	49 (25.5%)	56 (20.1%)	0.163
Hyperlipidemia	52 (38.2%)	33 (30.8%)	0.230	70 (36.5%)	83 (29.7%)	0.127
History of IHD	12 (8.8%)	18 (16.8%)	0.060	20 (10.4%)	33 (11.8%)	0.634
History of stroke	3 (2.2%)	5 (4.7%)	0.306	9 (4.7%)	31 (11.1%)	0.014
Imaging biomarkers						
Moderate to severe WMH	34 (25.0%)	32 (29.9%)		84 (43.8%)	193 (69.2%)	
Aβ-positivity^††^	—	—		99 (51.6%)	165 (59.1%)	

^†^ *APOE* genotyping was performed in 664 (93.0%) of the 714 participants in this study.

^††^Aβ PET was performed in cognitively impaired patients (N = 471) in this study.

Statistical analyses were performed with Chi-square, Fisher’s exact or Student’s t-tests.

Abbreviations: N = number; SD = standard deviation; DELC = diagonal earlobe crease; CN = cognitively normal; *APOE* = apolipoprotein E; MMSE = mini-mental state examination; CVD = cardiovascular disease; DM = diabetes mellitus; IHD = ischemic heart disease; WMH = white matter hyperintensities; Aβ = amyloid-beta.

Values are mean (SD) or N (%).

Detailed demographic and clinical characteristics of 714 study participants according to diagnosis are shown in Supplementary Table 1.

### The presence of DELC according to cognition and diagnosis

**Figure 1:**
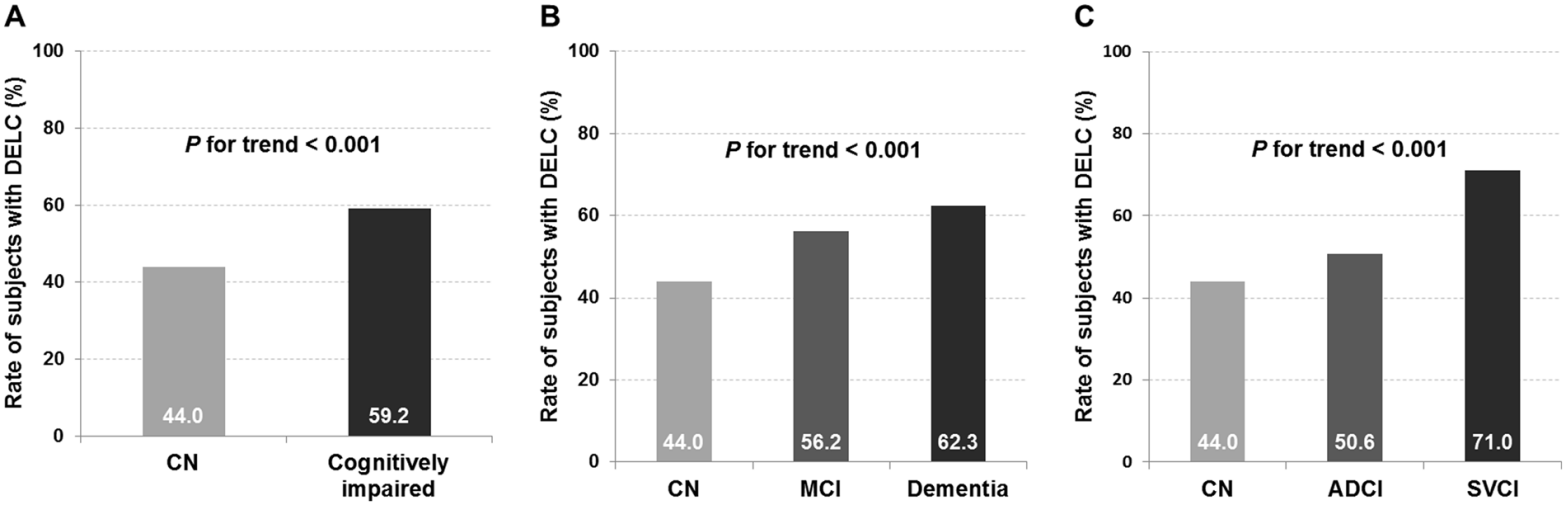
The linear trends of proportion of subjects with DELC according to cognition and diagnosis. DELC = diagonal earlobe crease; CN = cognitively normal; MCI = mild cognitive impairment; ADCI = Alzheimer’s disease-related cognitive impairment; SVCI = subcortical vascular cognitive impairment.

The proportion of subjects with DELC showed positive linear trends associated with cognitive and diagnostic status (Fig. 1). Specifically, DELC was more common in cognitively impaired than in CN participants. DELC was also more common in demented than in mild cognitive impairment (MCI) participants, and more common in MCI than in CN. DELC was more common in subcortical vascular cognitive impairment (SVCI) than in Alzheimer’s disease-related cognitive impairment (ADCI) participants, and more common in ADCI than in CN. Regression analyses of the presence of DELC according to cognitive and diagnostic groups are presented in Table 2. Cognitively impaired patients had more frequent DELC than CN individuals on both univariate and multivariate analyses. Compared with CN individuals, patients with MCI and dementia had significantly more frequent DELC in univariate analyses. However, this relationship remained significant in patients with dementia (OR 1.81, 95% CI 1.24–2.65, *P* = 0.002), but not patients with MCI, on multivariate analysis. Furthermore, patients with SVCI had more frequent DELC than CN individuals in multivariate analysis (OR 2.38, 95% CI 1.56–3.63, *P* = 0.002). There was no significant difference in the frequency of DELC between CN individuals and patients with ADCI.

**Table 2:** Regression analyses of the presence of DELC according to cognition and diagnosis.

	N	Univariate analyses		Multivariate analyses^†^	
		Presence of DELC		Presence of DELC	
		OR (95% CI)	*P*-value	OR (95% CI)	*P*-value
CN	243	Reference		Reference	
Cognitively impaired	471	1.85 (1.35–2.53)	<0.001	1.57 (1.13–2.17)	0.007
CN	243	Reference		Reference	
MCI	235	1,63 (1.14–2.34)	0.008	1.37 (0.94–2.00)	0.103
Dementia	236	2.10 (1.46–3.03)	<0.001	1.81 (1.24–2.65)	0.002
CN	243	Reference		Reference	
ADCI	271	1.30 (0.92–1.84)	0.140	1.23 (0.86–1.76)	0.268
SVCI	200	3.11 (2.09–4.63)	<0.001	2.38 (1.56–3.63)	<0.001

^†^Multivariate logistic regression analyses were performed after adjusting for age, sex, hypertension, diabetes mellitus, hyperlipidemia, and history of stroke and ischemic heart disease.

Abbreviations: DELC = diagonal earlobe crease; N = number; OR = odds ratio; CI = confidence interval; CN = cognitively normal; MCI = mild cognitive impairment; ADCI = Alzheimer’s disease-related cognitive impairment; SVCI = subcortical vascular cognitive impairment.

### WMH, Aβ-positivity, and DELC in cognitively impaired patients

In cognitively impaired patients, the frequencies of Aβ-positivity and of moderate to severe WMH were 56.1% and 58.8%, respectively (Supplementary Table 1). To evaluate the impacts that degree of WMH and Aβ-positivity had on the presence of DELC, we classified the 471 cognitively impaired patients into four groups, based on Aβ-positivity (Aβ+/−) and the presence of moderate to severe WMH (WMH+/−).

Compared with the WMH−/Aβ− group, the WMH−/Aβ+, WMH+/Aβ−, and WMH+/Aβ+ groups each had significantly more frequent DELC on both univariate and multivariate analyses (Table 3). In particular, the WMH+/Aβ+ group exhibited significantly more frequent DELC on multivariate analyses (OR 7.32, 95% CI 3.35–16.01, *P* < 0.001).

**Table 3:** Regression analyses of the presence of DELC in cognitively impaired patients based on WMH category and Aβ-positivity.

	N	Univariate analyses		Multivariate analyses^†^	
		Presence of DELC		Presence of DELC	
		OR (95% CI)	*P*-value	OR (95% CI)	*P*-value
WMH−/Aβ− group	57	Reference		Reference	
WMH−/Aβ+ group	137	2.39 (1.24–4.61)	0.010	2.94 (1.39–6.20)	0.005
WMH+/Aβ− group	150	4.31 (2.23–8.32)	<0.001	3.29 (1.59–6.82)	0.001
WMH+/Aβ+ group	127	7.29 (3.63–14.63)	<0.001	7.32 (3.35–16.01)	<0.001

^†^Multivariate logistic regression analyses were performed after adjusting for age, sex, hypertension, diabetes mellitus, hyperlipidemia, history of stroke and ischemic heart disease, and *APOE* ε4 status.

Abbreviations: WMH = white matter hyperintensities; Aβ = amyloid-beta; DELC = diagonal earlobe crease; OR = odds ratio; CI = confidence interval; N = number; *APOE* = apolipoprotein E; WMH−/Aβ− = patients with minimal WMH and Aβ-negative; WMH−/Aβ+ = patients with minimal WMH and Aβ-positive; WMH+/Aβ− = patients with moderate to severe WMH and Aβ-negative; WMH+/Aβ+ = patients with moderate to severe WMH and Aβ-positive.

## Discussion

In this study we observed several interesting findings that have not been previously reported, such as the frequency of DELC in cognitively impaired patients, and the associations between DELC, CSVD, and Aβ-positivity. The major findings of the present study were as follows. First, compared with CN individuals, cognitively impaired patients more frequently exhibited DELC, and this difference was strongest in patients with dementia or SVCI. Second, among cognitively impaired patients, degree of WMH and Aβ-positivity independently predicted the presence of DELC. Taken together, our findings suggest that DELC can serve as a useful supportive sign, not only for the diagnosis of cognitive impairment, but also for potential CSVD and Aβ-positivity.

Our first major finding was that DELC was more common in cognitively impaired patients than in individuals with normal cognition. DELC has been reported to be associated with CAD^1,2,5,12^, and with cardiovascular risk factors such as hypertension, diabetes, hyperlipidemia, and metabolic syndrome^6–8^. Furthermore, previous studies with a large number of healthy subjects have reported that DELC becomes more common with age^3,7^. Although the cognitively impaired patients in this study were older than the CN individuals, the increased frequency of DELC for cognitively impaired patients relative to CN individuals remained after adjustment for age, sex, and conventional cardiovascular risk factors known to be associated with DELC. In addition, we found that DELC was more common in patients with dementia or SVCI compared with CN individuals. Therefore, our findings suggest that DELC seems to be a marker of cognitive impairment, especially in patients with dementia or SVCI.

Another noteworthy finding in this study was that WMH burden and Aβ-positivity independently predicted the presence of DELC in cognitively impaired patients. Interestingly, the combination of both moderate to severe WMH and Aβ-positivity was most strongly associated with DELC, compared with the presence of only one or neither of these imaging markers. Several previous studies have reported that the presence of DELC may predict asymptomatic peripheral arterial disease, arterial stiffness, and subclinical atherosclerosis^13–16^. Moreover, a recent prospective study demonstrated a significant association between DELC and the development of ischemic stroke^17^. To date, however, no visible signs have been shown to predict CSVD burden or Aβ-positivity in cognitively impaired patients. Importantly, our data suggest that DELC may serve as a visible sign for CSVD and Aβ accumulation in clinical practice.

The mechanisms underlying the relationships among DELC, CSVD, and Aβ accumulation have not been fully investigated. Previous studies showing that DELC is a risk marker for CAD have suggested a common mechanism of microvascular changes related to subclinical atherosclerosis, as both the earlobe and myocardium are supplied by end-arteries with few collaterals^2,5,9,10^. There may also be a shared pathophysiology for DELC and CSVD in terms of the location of arteriosclerosis, as CSVD is thought to occur between the perforating cerebral arterioles or capillaries in the brain^18,19^, and DELC is known to occur where the branches of the superficial temporal and posterior auricular arteries meet^20,21^. Elastin degeneration could also potentially explain the relationship between DELC and CSVD. Specifically, elastin degeneration in the skin may reflect changes in vessel walls with similar elastic properties^5,22^. This is supported by previous studies showing an association between DELC and arterial stiffness^14,15^. Furthermore, Aβ is also known to alter the neurovascular unit in the brain^23,24^, and several previous studies have shown that Aβ and CSVD interact with each other^25,26^. Therefore, like CSVD, Aβ might affect arteriosclerosis, which in turn may lead to DELC and CSVD.

The strengths of this study include a large sample size and relatively sophisticated measurements of DELC compared with previous studies. However, some limitations should be considered when interpreting these results. First, our study was cross-sectional, precluding claims of causality. Second, while WMH burden was assessed using structured semi-quantitative methods, this visual rating scale may not fully reflect the degree of CSVD. Finally, since we only collected Aβ PET scans on cognitively impaired patients, our interpretations regarding amyloid accumulation in normal subjects are limited. Together our results suggest that DELC could be a useful supportive sign for the presence of cognitive impairment, CSVD, and Aβ-positivity, but further investigations are required to identify underlying mechanisms of their relationships.

## Methods

### Study participants

A total of 506 patients with cognitive impairment were included from two prospective studies using Aβ PET and brain MRI scans at the Samsung Medical Center (Seoul, Korea): (1) 114 patients with ADCI and 137 with SVCI who underwent ^11^C-Pittsburgh compound B (PiB) PET scans from September 2008 to September 2011; and (2) 177 patients with ADCI and 78 with SVCI who underwent ^18^F-florbetaben PET scans from August 2015 to September 2016. Patients who were clinically diagnosed with either amnestic MCI or probable AD dementia were grouped as ADCI. Patients who were clinically diagnosed with either subcortical vascular MCI or subcortical vascular dementia were grouped as SVCI. Clinical diagnosis was established at a multi-disciplinary conference applying standard research criteria for MCI and dementia syndromes^27,28^. Specifically, patients with SVCI met the following criteria^29^: (1) a subjective cognitive complaint from either the patient or caregiver, (2) an objective cognitive impairment below the 16th percentile in any domain including attention, language, visuospatial, memory, or frontal function on neuropsychological tests^30^, (3) significant ischemia on brain MRI, defined as periventricular WMH ≥ 10 mm and deep WMH ≥ 25 mm, as modified from Fazekas’ ischemia criteria and as described in previous studies^31,32^, and (4) focal neurologic symptoms or signs.

We consecutively recruited 357 CN individuals from the Memory Clinic at the Samsung Medical Center from January 2013 to December 2014. All CN individuals had no objective cognitive dysfunction on detailed neuropsychological testing, and underwent brain MRI. Exclusion criteria included history of traumatic brain injury, cortical stroke, seizure, brain surgery, and current systemic medical disease that could affect cognition. Since it has been shown that the prevalence of DELC increases with age^3^, we included 261 CN individuals in this study who were 60 years of age or older.

We excluded 53 participants for whom we could not discriminate the presence of DELC on 3-dimensional (3D) reconstructed brain MRI scans because of head motion, MRI blurring, or folded earlobes. The final sample of 714 participants included 271 ADCI patients, 200 SVCI patients, and 243 CN individuals. Brain MRI confirmed the absence of structural lesions including territorial cerebral infarction, brain tumors, hippocampal sclerosis, and vascular malformation. Blood tests included a complete blood count, blood chemistry, vitamin B12/folate, syphilis serology, and thyroid function tests. Apolipoprotein E (*APOE*) genotyping was performed in 664 (93.0%) of the 714 participants.

### Standard protocol approvals, registrations, and patient consents

We obtained written informed consent from each participant. This study was approved by the Institutional Review Board at the Samsung Medical Center. In addition, all methods were carried out in accordance with the approved guidelines.

### Brain MRI scans

All study participants underwent a 3D volumetric brain MRI scan. An Achieva 3.0-Tesla MRI scanner (Philips, Best, the Netherlands) was used to acquire 3D T1 Turbo Field Echo MRI data from 714 participants using the following imaging parameters: sagittal slice thickness of 1.0 mm with 50% overlap; no gap; repetition time of 9.9 ms; echo time of 4.6 ms; flip angle of 8°; and matrix size of 240 × 240 pixels reconstructed to 480 × 480 over a field of view of 240 mm.

### Evaluation of the presence of DELC on 3D-reconstructed MRI scans

**Figure 2:**
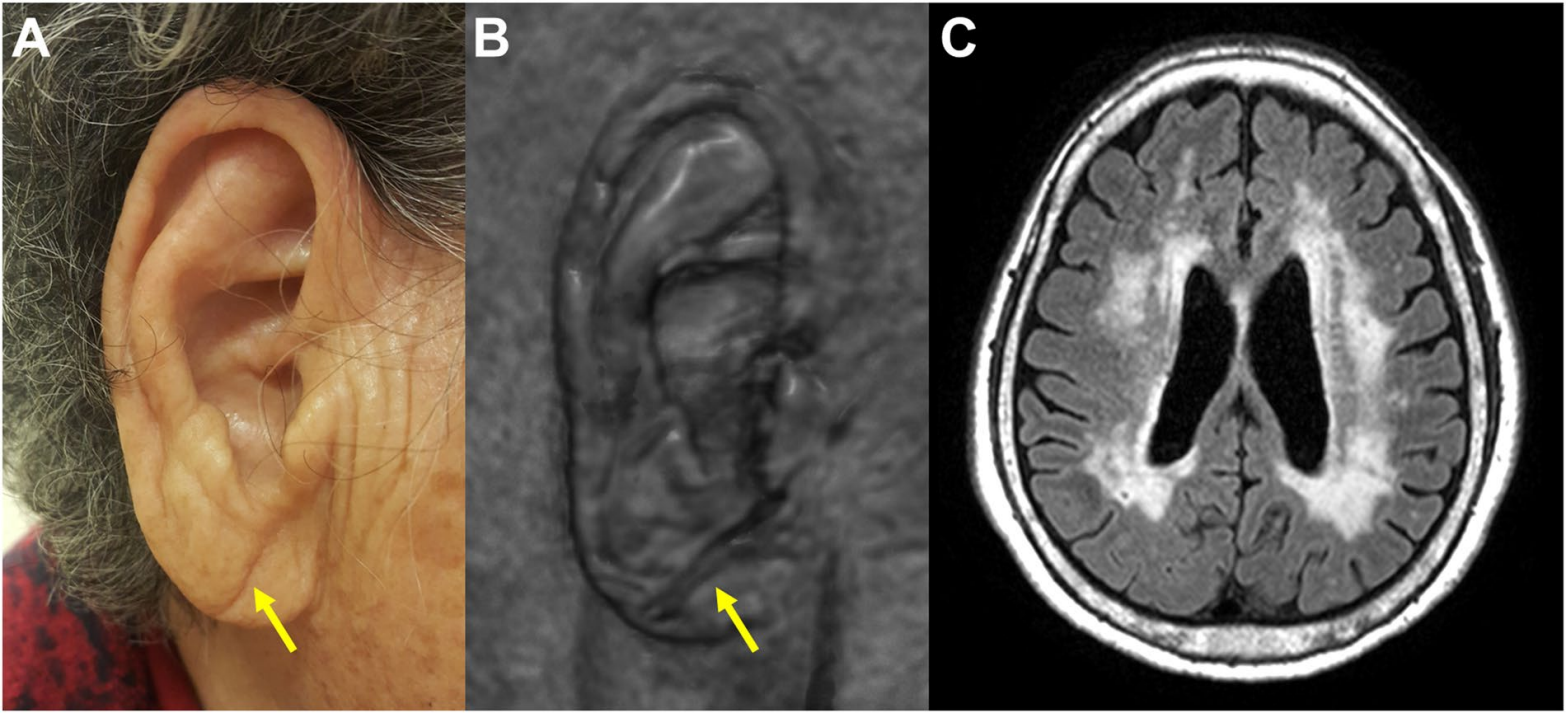
DELC **(**arrows) on photograph (**A**) and 3D-reconstructed MRI (**B**) in a cognitively impaired patient. The patient’s brain MRI shows severe WMH on FLAIR imaging (**C**). DELC = diagonal earlobe crease; MRI = magnetic resonance imaging; WMH = white matter hyperintensities; FLAIR = fluid-attenuated inversion recovery.

The ear lobes of study participants were assessed using 3D-reconstructed volumetric brain MRI scans. We used the MRIcroGL software (version 12 June 2015, freeware, Chris Rorden) to create a 3D model of the head^33^. We confirmed the accuracy and excellent quality of reconstructed images by comparing them with actual participants’ photographs in our clinic (Supplementary Figure 1). The typical DELC was defined as a wrinkle-like line extending diagonally from the tragus across the lobule to the rear edge of the auricle of the ear. We visually checked for the presence of a distinct crease on each earlobe (of complete length and moderate to severe depth), according to a previous study^34^. If no distinct crease was visible on either earlobe, the participant was assigned to the ‘No DELC’ group; if a distinct crease was observed on one or both earlobes, then the participant was assigned to the ‘DELC’ group. The 3D-reconstructed images of all study participants were examined by 2 trained observers who were blinded to participants’ diagnoses and clinical information. The observers had an overall inter-observer agreement of 91.7% for the presence of DELC. A representative DELC image and MRI of a cognitively impaired patient are shown in Fig. 2.

### WMH visual rating scale

We used a modified Fazekas scale for visual rating of WMH^31,32^. On this scale, periventricular WMH were classified as P1 (cap or band < 5 mm), P2 (5 mm ≤ cap or band < 10 mm), and P3 (cap or band ≥ 10 mm); deep WMH were classified into D1 (maximum diameter of deep white matter lesion < 10 mm), D2 (10 mm ≤ lesion < 25 mm), and D3 (≥25 mm). The intra-class correlation coefficients for inter-rater reliability of the WMH visual rating scale were excellent (Cohen’s kappa = 0.73–0.91)^35^. The WMH visual rating scale also correlated well with automated measurements of WMH volume^36^. WMH ratings were combined to give a final classification of minimal (combinations of D1 with P1 [D1P1] and D1 with P2 [D1P2]), moderate (D2P1, D3P1, D2P2, D3P2, D1P3, and D2P3) or severe (D3P3). We considered moderate to severe degree of WMH as the presence of WMH in the present study.

### Aβ PET acquisition and analysis

#### ^11^C-PiB PET

^11^C-PiB PET scanning was performed at the Samsung Medical Center using a Discovery STe PET/Computed tomography (CT) scanner (GE Medical Systems, Milwaukee, WI, USA) operated in 3D scanning mode (35 slices of 4.25 mm thickness spanning the entire brain). ^11^C-PiB was injected into an antecubital vein as a bolus at a mean dose of 420 MBq (range 259–550 MBq). A CT scan was performed for attenuation correction at 60 minutes after the injection. A 30-minute emission static PET scan was then initiated. Data processing was performed with SPM version 2 using Matlab 6.5 (MathWorks, Natick, MA). ^11^C-PiB PET images were co-registered to each individual’s MRI, which were normalized to a T1-weighted MRI template.

We used the cerebral cortical region to cerebellum uptake ratio, which is identical to the standardized uptake value ratio (SUVR) to measure PiB retention. The cerebellar cortex was used as a reference region, since there is little specific binding of PiB in postmortem samples of cerebellar cortex even among those with AD at autopsy^37^. We chose 28 cerebral cortical VOIs for this study, from regions in bilateral frontal, posterior cingulate, parietal, lateral temporal, and occipital cortices (Supplementary Table 2). The global PiB uptake ratio was calculated from the volume-weighted average SUVR of 28 bilateral cerebral cortical VOIs.

Patients were considered Aβ-positive if their global PiB SUVR was 1.5 or higher^38^. The detailed methods for ^11^C-PiB PET scanning and the calculation of global PiB retention ratio are described in previous studies^38,39^.

#### ^18^F-florbetaben PET

Patients underwent ^18^F-florbetaben PET scanning at the Samsung Medical Center using the same type of scanner used for the ^11^C-PiB PET scanning in 3D scanning mode (47 slices of 3.3 mm thickness spanning the entire brain). CT images were acquired using a 16-slice helical CT (140 KeV, 80 mA; 3.75 mm section width) for attenuation correction. For ^18^F-florbetaben PET, a 20-minute emission PET scan with dynamic mode (consisting of 4 × 5 min frames) was performed 90 minutes after injection of 300 MBq ± 20% ^18^F-florbetaben. 3D PET images were reconstructed in a 128 × 128 × 48 matrix with a 2 × 2 × 3.27 mm voxel size using the ordered-subsets expectation maximization algorithm (iteration = 4 and subset = 20).

All images were assessed visually by trained experts (nuclear medicine physicians) according to brain amyloid plaque load (BAPL) scoring^40^. Specifically, experts used a regional cortical tracer uptake (RCTU) scoring system (RCTU 1: no tracer uptake; RCTU 2: moderate tracer uptake; and RCTU 3: pronounced tracer uptake) in 4 brain areas: lateral temporal cortex, frontal cortex, posterior cingulate cortex/precuneus, and parietal cortex. An RCTU score of 1 in each brain region led to a BAPL score of 1, while an RCTU score of 2 but no RCTU score 3 in any brain region led to a BAPL score of 2. An RCTU score of 3 in any of the 4 brain regions led to a BAPL score of 3. The resulting scores were condensed into a binary interpretation (BAPL score 1: Aβ-negative; BAPL score 2 or 3: Aβ-positive).

### Statistical analysis

Continuous variables were presented as means ± standard deviation (SD) and were compared using Student’s t-test. Categorical variables were compared using the Chi-square test or Fisher’s exact test. The linear by linear association was applied for trend analyses. Univariate logistic regression analyses were performed to determine whether moderate to severe degree of WMH was associated with the presence of DELC. In addition, multivariate logistic regression analyses were performed after adjusting for age, sex, hypertension, diabetes mellitus, hyperlipidemia, and history of stroke and ischemic heart disease. To determine whether moderate to severe degree of WMH and Aβ-positivity were associated with the presence of DELC, we further adjusted for *APOE* ε4 status. Statistical significance was set at *P* < 0.05. Statistical analyses were conducted using SPSS 20 (SPSS Inc., Chicago, IL, USA).

## Electronic supplementary material

Dataset 1
