# Supplementary material for: Diagonal Earlobe Crease is a Visible Sign for Cerebral Small Vessel Disease and Amyloid-β
Source: Sci Rep. 2017 Oct 17;7:13397. doi: 10.1038/s41598-017-13370-8 (PMC5645376; doi:10.1038/s41598-017-13370-8)
Supplement: Supplementary file 1 — Dataset 1 [file 41598_2017_13370_MOESM1_ESM.doc]

**Diagonal Earlobe Crease is a Visible Sign for**

**Cerebral Small Vessel Disease and Amyloid-β**

Jin San Lee,^1-3^ Seongbeom Park,^1,2^ Hee Jin Kim,^1,2^ Yeshin Kim,^1,2^ Hyemin Jang,^1,2^ Ko Woon Kim,^4^ Hak Young Rhee,^5^ Sung Sang Yoon,^3^ Kyoung Jin Hwang,^3^ Key-Chung Park,^3^ Seung Hwan Moon,^6^ Sung Tae Kim,^7^ Samuel N. Lockhart,^8,9^ Duk L. Na,^1,2,10^ Sang Won Seo^1,2,10,11^

^1^ Department of Neurology, Samsung Medical Center, Sungkyunkwan University School of Medicine, Seoul 06351, Korea

^2^ Neuroscience Center, Samsung Medical Center 06351, Seoul, Korea

^3^ Department of Neurology, Kyung Hee University Hospital, Seoul, Korea

^4^ Department of Neurology, Chonbuk National University Hospital, Jeonju, Korea

^5^ Department of Neurology, Kyung Hee University Hospital at Gangdong, Kyung Hee University School of Medicine, Seoul, Korea

^6^ Departments of Nuclear Medicine, Samsung Medical Center, Sungkyunkwan University School of Medicine, Seoul 06351, Korea

^7^ Department of Radiology, Samsung Medical Center, Sungkyunkwan University School of Medicine, Seoul 06351, Korea

^8^ Helen Wills Neuroscience Institute, University of California Berkeley, Berkeley, CA 94720, USA

^9^ Department of Internal Medicine, Division of Gerontology and Geriatric Medicine, Wake Forest School of Medicine, Winston-Salem, NC 27157, USA

^10^ Department of Health Sciences and Technology, ^11^ Clinical Research Design and Evaluation, SAIHST, Sungkyunkwan University, Seoul 06351, Korea

**Corresponding author**

Sang Won Seo, MD, PhD

Department of Neurology, Samsung Medical Center, Sungkyunkwan University School of Medicine, 81 Irwon-ro, Kangnam-ku, Seoul 06351, Korea

Tel: +82-2-3410-1233, Fax: +82-2-3410-0052, E-mail address: sangwonseo@empal.com

**Running title:** Diagonal earlobe crease and dementia

**Supplementary data:** Supplementary Tables 1 and 2, and Supplementary Figure 1

**Supplementary Table 1** Demographic characteristics of the study participants according to diagnosis (N = 714)

|  | **CN** | **Cognitively impaired** |  | **ADCI** |  |  |  | **SVCI** |  |
| --- | --- | --- | --- | --- | --- | --- | --- | --- | --- |
|  |  |  | **Total** | **aMCI** | **AD** |  | **Total** | **svMCI** | **SVaD** |
| **Total, N** | 243 | 471 | 271 | 136 | 135 |  | 200 | 99 | 101 |
| **Age, years** | 70.8 (7.0)^a,b^ | 72.9 (8.4)^a^ | 71.3 (9.0)^c^ | 72.1 (8.1) | 70.5 (9.7) |  | 75.1 (6.9)^b,c^ | 74.4 (6.4) | 75.7 (7.2) |
| **Male** | 68 (28.0)^a,b^ | 192 (40.8)^a^ | 123 (45.4)^b,c^, | 67 (49.3) | 56 (41.5) |  | 69 (34.5)^c^ | 36 (36.4) | 33 (32.7) |
| **Education, years** | 10.4 (4.9)^a,b^ | 10.7 (5.4) | 12.3 (4.8)^a,c^ | 13.0 (4.5) | 11.6 (5.1) |  | 8.5 (5.4)^b,c^ | 9.2 (5.6) | 7.8 (5.2) |
| ***APOE* genotype*** |  |  |  |  |  |  |  |  |  |
| ***APOE*** **ε2 present** | 32 (14.7)^a,b^ | 41 (9.2)^a^ | 20 (8.1)^b^ | 10 (8.1) | 10 (8.0) |  | 21 (10.6) | 11 (11.1) | 10 (10.0) |
| ***APOE* ε4 present** | 38 (15.6)^a,b,c^ | 173 (38.7)^a^ | 115 (46.4)^b,d^, | 47 (38.2) | 68 (54.4) |  | 58 (29.1)^c,d^ | 26 (26.3) | 32 (32.0) |
| **MMSE** | 28.3 (2.0)^a,b,c^ | 22.7 (5.4)^a^ | 22.7 (5.5)^b^ | 26.4 (2.9) | 19.1 (5.1) |  | 22.7 (5.2)^c^ | 25.7 (3.4) | 19.6 (4.9) |
| **CVD risk factors** |  |  |  |  |  |  |  |  |  |
| **Hypertension** | 113 (46.5)^a,b^ | 275 (58.4)^a^ | 124 (45.8)^c^ | 58 (42.6) | 66 (48.9) |  | 151 (75.5)^b,c^ | 73 (73.7) | 78 (77.2) |
| **DM** | 45 (18.5) | 105 (22.3) | 55 (20.3) | 25 (18.4) | 30 (22.2) |  | 50 (25.0) | 27 (27.3) | 23 (22.8) |
| **Hyperlipidemia** | 85 (35.0) | 153 (32.5) | 86 (31.7) | 44 (32.4) | 42 (31.1) |  | 67 (33.5) | 31 (31.3) | 36 (35.6) |
| **History of IHD** | 30 (12.3) | 53 (11.3) | 26 (9.6) | 16 (11.8) | 10 (7.4) |  | 27 (13.5) | 21 (21.2) | 6 (5.9) |
| **History of stroke** | 8 (3.3)^a,b^ | 40 (8.5)^a^ | 6 (2.2)^c^ | 1 (0.7) | 5 (3.7) |  | 34 (17.0)^b,c^ | 14 (14.1) | 20 (19.8) |
| **Imaging biomarkers** |  |  |  |  |  |  |  |  |  |
| **Moderate to severe degree of WMH** | 66 (27.2)^a,b^ | 277 (58.8)^a^ | 77 (28.4)^c^ | 35 (25.7) | 42 (31.1) |  | 200 (100.0)^b,c^ | 99 (100.0) | 101 (100.0) |
| **Aβ-positivity** | - | 264 (56.1) | 195 (72.0) | 72 (52.9) | 123 (91.1) |  | 69 (34.5) | 31 (31.3) | 38 (37.6) |

Values are mean (SD) or N (%).

* *APOE* genotyping was performed in 664 (93.0%) of the 714 participants in this study.

Abbreviations: N = number; SD = standard deviation; CN = cognitively normal; ADCI = Alzheimer’s disease-related cognitive impairment; aMCI = amnestic mild cognitive impairment; AD = Alzheimer’s disease; SVCI = subcortical vascular cognitive impairment; svMCI = subcortical vascular mild cognitive impairment; SVaD = subcortical vascular dementia; *APOE* = apolipoprotein E; MMSE = mini-mental state examination; CVD = cardiovascular disease; DM = diabetes mellitus; IHD = ischemic heart disease; WMH = white matter hyperintensities; Aβ = amyloid-beta.

**Supplementary Table 2** Twenty-eight cerebral cortical volumes of interest to measure ^11^C-Pittsburgh compound B retention

| **Frontal** | Superior and middle frontal gyri, medial part of the superior frontal gyrus, opercular part of the inferior frontal gyrus, triangular part of the inferior frontal gyrus, supplementary motor area, orbital part of the superior, middle, and inferior orbital frontal gyri, rectus and olfactory cortex |
| --- | --- |
| **Posterior cingulate** |  |
| **Parietal** | Superior and inferior parietal, supramarginal and angular gyri, and precuneus |
| **Lateral temporal** | Superior, middle, and inferior temporal gyri, and Heschl’s gyri |
| **Occipital** | Superior, middle, and inferior occipital gyri, cuneus, calcarine fissure, and lingual and fusiform gyri |

A total of 28 cortical volumes of interest were chose from both the left and right cerebral hemisphere using the automated anatomical labeling atlas.

**Figure Legends**

**Supplementary Figure 1** The comparison between actual participants’ photographs (left) and 3D-reconstructed images (right), for a participant with no DELC (A) and participants with DELC (B and C).

DELC = diagonal earlobe crease.

**
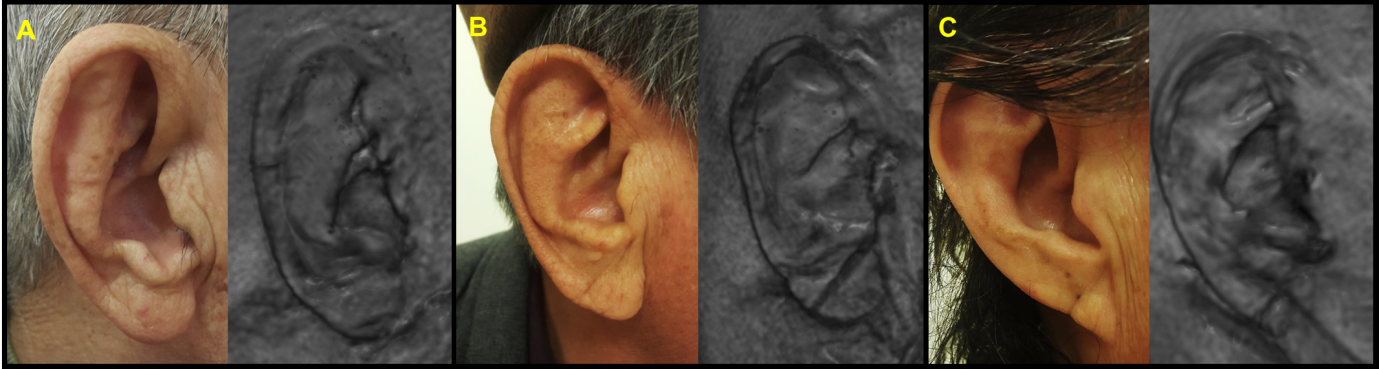
**

**Supplementary Figure 1** The comparison between actual participants’ photographs (left) and 3D-reconstructed images (right), for a participant with no DELC (A) and participants with DELC (B and C).

DELC = diagonal earlobe crease.
